# Contribution of Cytidine Deaminase to Thymidylate Biosynthesis in Trypanosoma brucei: Intracellular Localization and Properties of the Enzyme

**DOI:** 10.1128/mSphere.00374-19

**Published:** 2019-08-07

**Authors:** Ana Moro-Bulnes, Víctor M. Castillo-Acosta, Maria Valente, Juana Carrero-Lérida, Guiomar Pérez-Moreno, Luis Miguel Ruiz-Pérez, Dolores González-Pacanowska

**Affiliations:** a Instituto de Parasitología y Biomedicina López-Neyra, Consejo Superior de Investigaciones Científicas, Parque Tecnológico de Ciencias de la Salud, Armilla, Granada, Spain; University of Georgia

**Keywords:** *Trypanosoma brucei*, cytidine deaminase, pyrimidine metabolism, thymidylate biosynthesis

## Abstract

Cytidine deaminase (CDA) is a pyrimidine salvage enzyme that catalyzes cytidine and deoxycytidine hydrolytic deamination to yield uridine and deoxyuridine. Here we report the biochemical characterization of Trypanosoma brucei CDA as an enzyme within the tetrameric class of the CDA family that efficiently deaminates cytidine, deoxycytidine, and the nucleoside analogue 5-methyl-2′-deoxycytidine. In line with previous studies, we show that RNA interference (RNAi)-mediated CDA depletion impairs T. brucei proliferation when grown in pyrimidine-deficient medium, while supplementation with thymidine or deoxyuridine restores growth, further underscoring the role of this enzyme in providing deoxyuridine for dUMP formation via thymidine kinase, the substrate required for *de novo* thymidylate biosynthesis. This observation contrasts with the existence in T. brucei of a dimeric deoxyuridine 5′-triphosphate nucleotidohydrolase (dUTPase), an essential enzyme that can produce dUMP via the hydrolysis of dUTP/dUDP. Thus, T. brucei dUTPase-null mutants are thymidine auxotrophs, suggesting that dUTPase might have a role in providing dUMP for thymidylate biosynthesis. We show that overexpression of human dCMP deaminase (DCTD), an enzyme that provides directly dUMP through dCMP deamination, does not reverse the lethal phenotype of dUTPase knockout cells, which further supports the notion that in T. brucei, CDA is uniquely involved in providing dUMP, while the main role of dUTPase would be the withdrawal of the excess of dUTP to avoid its incorporation into DNA. Furthermore, we report the mitochondrial localization of CDA, highlighting the importance of this organelle in pyrimidine metabolism.

**IMPORTANCE** Cytidine deaminases (CDAs) catalyze the hydrolytic deamination of cytidine and deoxycytidine in the pyrimidine salvage pathway. In kinetoplastids, pyrimidine metabolism has been extensively studied as a source of potential drug targets, given the fact that many of the enzymes of the pathway are essential. Thymidylate (dTMP) synthesis in Trypanosoma brucei exhibits unique characteristics. Thus, it has been suggested that the production of dUMP, the substrate for dTMP formation, is solely dependent on cytidine deaminase and thymidine kinase. Here we characterize recombinant T. brucei CDA (TbCDA) and present evidence that indeed the alternative route for dUMP formation via deoxyuridine 5′-triphosphate nucleotidohydrolase does not have a prominent role in *de novo* dTMP formation. Furthermore, we provide a scheme for the compartmentalization of dTMP biosynthesis, taking into account the observation that CDA is located in the mitochondrion, together with available information on the intracellular localization of other enzymes involved in the dTTP biosynthetic pathway.

## Abstract (precis)

Cytidine deaminases (CDAs) catalyze the hydrolytic deamination of cytidine and deoxycytidine in the pyrimidine salvage pathway. In kinetoplastids, pyrimidine metabolism has been extensively studied as a source of potential drug targets, given the fact that many of the enzymes of the pathway are essential. Thymidylate (dTMP) synthesis in Trypanosoma brucei exhibits unique characteristics. Thus, it has been suggested that the production of dUMP, the substrate for dTMP formation, is solely dependent on cytidine deaminase and thymidine kinase. Here we characterize recombinant T. brucei CDA (TbCDA) and present evidence that indeed the alternative route for dUMP formation via deoxyuridine 5′-triphosphate nucleotidohydrolase does not have a prominent role in *de novo* dTMP formation. Furthermore, we provide a scheme for the compartmentalization of dTMP biosynthesis, taking into account the observation that CDA is located in the mitochondrion, together with available information on the intracellular localization of other enzymes involved in the dTTP biosynthetic pathway.

## INTRODUCTION

Trypanosoma brucei is an extracellular protozoan parasite within the Kinetoplastida order and the causative agent of African trypanosomiasis. The subspecies T. brucei *rhodesiense* and T. brucei *gambiense* are responsible for human African trypanosomiasis (HAT), or sleeping sickness, while T. brucei *brucei* infects only domestic and wild animals. Even though several drugs for treatment of the first and second stages of HAT are currently available, severe adverse side effects, poor efficacy, and complex treatment (1, 2) are some of the reasons why there is a real need for the development of new drugs.

A well-controlled pool of deoxyribonucleotides (deoxynucleoside triphosphates [dNTPs]) is crucial for DNA biosynthesis and replication and subsequently for cell integrity. dUMP formation for thymidylate (dTMP) synthesis is essential for T. brucei survival and occurs through phosphorylation of deoxyuridine by the action of T. brucei thymidine kinase (TbTK) (3, 4). The major source of deoxyuridine (dUrd) for dUMP formation has been described as coming from deoxycytidine (dCtd) deamination via T. brucei cytidine deaminase (TbCDA) (3). Impairment of dTMP generation by downregulation of TbTK leads to decreased dTTP levels and the so-called “thymineless death” (5). In addition to TbTK, dihydrofolate reductase-thymidylate synthase (TbDHFR-TS), a dimeric bifunctional enzyme responsible for the reductive methylation of dUMP to dTMP, is also essential for parasite survival in the absence of extracellular thymidine (dThd) (3, 4, 6).

The essential character of TbTK resides in the observation that T. brucei lacks a dCMP deaminase (DCTD) and subsequently a route for dUMP formation through dCMP deamination. However, dUMP can also be formed through the hydrolysis of dUTP via T. brucei deoxyuridine 5′-triphosphate nucleotidohydrolase (TbdUTPase), an enzyme that catalyzes the hydrolysis of both dUDP and dUTP to dUMP and P_i_ or PP_i_, respectively (7). This apparent redundancy poses questions regarding the role of TbdUTPase in dTMP biosynthesis. TbdUTPase has been shown to be essential (8), and knockout parasites are dThd auxotrophs and exhibit a hypermutator phenotype (7).

Cytidine deaminase (CDA) has been described in different organisms as a pyrimidine salvage pathway enzyme that catalyzes the hydrolytic deamination of cytidine (Ctd) and dCtd to uridine and dUrd, respectively, and is also capable of deaminating several nucleoside analogues used in cancer treatment, such as decitabine (5′-aza-2′-deoxycytidine) (9), gemcitabine (2′,2′-difluoro-2′-deoxycytidine) (10), or cytarabine (1-β-d-arabinofuranosylcytosine [Ara-C]) (11).

From the structural point of view, CDAs are divided in two different classes: the homodimeric CDAs, such as Escherichia coli CDA or Arabidopsis thaliana CDA, which consist of two identical monomers of approximately 32 kDa (12, 13), and the homotetrameric CDAs, such as Homo sapiens CDA or Bacillus subtilis CDA, which consist of four identical polypeptides of approximately 15 kDa (14, 15). In all cases, the molecular mass of the native enzyme is 60 to 64 kDa, and each subunit coordinates a zinc ion in the active site, which in dimeric CDAs like that of E. coli is formed with the contribution of residues from the so-called “broken active site” of the other monomer (12), whereas in tetrameric CDAs like that of B. subtilis, it is built through a series of intersubunit interactions (15–17).

Here we aim to study the contribution of TbCDA (3) and TbdUTPase (7, 8) to dUMP formation for dTMP synthesis. We report the biochemical characterization of TbCDA and the confirmation of its essential role in dUMP formation for dTMP synthesis via TbTK. On the other hand, we present data indicating that the essential character of TbdUTPase is mainly related to the maintenance of cellular dUTP concentrations, while TbCDA and TbTK would be the enzymes uniquely involved in dUMP production. A scheme for the intracellular compartmentalization of dTTP synthesis in T. brucei is proposed herein.

## RESULTS

### TbCDA belongs to the homotetrameric class of the CDA family and efficiently deaminates cytidine and deoxycytidine.

In an effort to characterize TbCDA (Tb927.9.3000), the coding sequence was cloned in pET28a in order to express a recombinant His tag fusion protein for further purification and kinetic analysis. TbCDA was efficiently expressed in a soluble form, and the protein was purified by metal-binding affinity chromatography followed by size exclusion chromatography (see Table S1 and Fig. S1 in the supplemental material). The final product was analyzed by SDS-PAGE, revealing a major band of 21.5 kDa with minor contaminants, which was identified as TbCDA by tryptic digestion and peptide fingerprinting.

10.1128/mSphere.00374-19.1 FIG S1 SDS-PAGE of the resulting purified TbCDA (21.5 kDa) after the desalting step with the PD-10 column. Gels were stained with Coomassie blue according to the manufacturer’s instructions. Download FIG S1, TIF file, 0.3 MB. Copyright © 2019 Moro-Bulnes et al. 2019 Moro-Bulnes et al. This content is distributed under the terms of the Creative Commons Attribution 4.0 International license.

10.1128/mSphere.00374-19.4 TABLE S1 Purification of recombinant TbCDA. Download Table S1, PDF file, 0.06 MB. Copyright © 2019 Moro-Bulnes et al. 2019 Moro-Bulnes et al. This content is distributed under the terms of the Creative Commons Attribution 4.0 International license.

**FIG 1:**
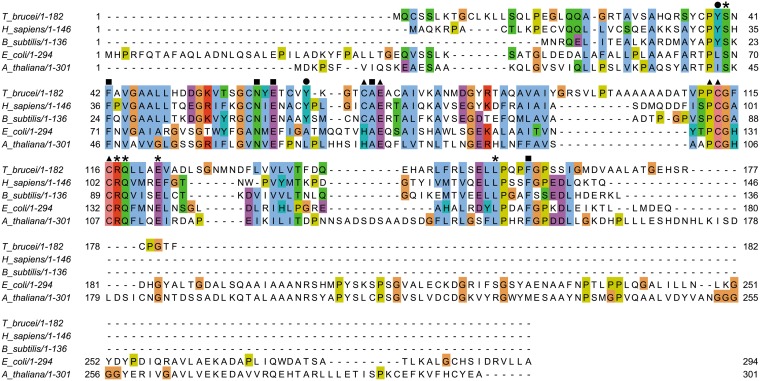
Sequence alignment of cytidine deaminase orthologs from several organisms, including Trypanosoma brucei (XP_803607.1), Homo sapiens (NP_001776.1), Bacillus subtilis (KOS70912.1), Escherichia coli (KHJ23847.1), and Arabidopsis thaliana (CAA06460.1). Amino acids involved in the zinc binding site are highlighted with solid triangles, residues responsible for substrate binding are denoted by solid squares, amino acids involved in domain-domain interactions are indicated by asterisks, and conserved tyrosine residues in tetrameric CDAs are marked with solid circles. The alignment was visualized with JalView according to the Clustal color scheme (http://www.jalview.org/help/html/colourSchemes/clustal.html).

Analysis of TbCDA by mass spectrometry revealed a major peak with an *m*/*z* value of 21,192.059, corresponding to the theoretical mass of the His tag fusion protein (21,334.2 Da), and a second peak with an *m*/*z* value of 42,568.411, which could correspond to the molecular mass of the dimer, although a tetrameric quaternary structure could not be discarded: given the fact that the interactions among the different subunits are ionic in nature and subsequently weak, the trimeric and tetrameric structures could have been unstable after ionization and therefore undetectable. The potential oligomeric structure of TbCDA was further analyzed by gel filtration, resulting in a major peak with a molecular mass of approximately 77.7 kDa, indicating that it is a member of the homotetrameric class of the CDA family. All the CDA superfamily members possess a zinc binding motif (H/C-X-E-X_24-36_P-C-X_2-4_C) (18, 19) with a histidine and two cysteine residues coordinating the zinc ion in the case of the homodimeric class, whereas in the homotetrameric class, the metal binding motif sequence consists of three cysteine residues (15). The alignment in Fig. 1 shows that indeed TbCDA exhibits the following zinc binding motif conserved residues: Glu72, analogous to the human CDA residue Glu67, which plays an important role in catalysis (20); Pro112; and the three characteristic cysteine residues (Cys70, Cys113, and Cys116) that coordinate the zinc ion. The alignment also shows the conservation of other important residues present in either tetrameric or dimeric CDAs, such as Phe42, Asn60, Glu62, Ala71, and Phe158, which are involved in substrate binding, as well as Ser40, Arg117, Gln118, Glu122, and Leu154, which are important for tetramer/dimer interactions (15). Tyr39 is conserved in tetrameric CDAs and is important for the stabilization of the quaternary structure (17). Likewise, the Tyr66 NH group is hydrogen bonded with the 5′-OH of the substrate (20) and is also conserved in tetrameric CDAs.

Table 1 shows the kinetic parameters for recombinant TbCDA determined for Ctd, dCtd, and 5-methyl-2′-deoxycytidine (5-Met-dCtd) as the substrates and measured as described previously (14). The enzyme exhibits Michaelis-Menten kinetics, and the *K_m_* values obtained for dCtd were similar to those of the human, E. coli, and *A. thaliana* CDAs (13). Table 2 presents the kinetic parameters previously published for human (14), E. coli (13), *A. thaliana* (13), and B. subtilis (16) CDAs. Neither dCMP nor dCTP was a substrate of the enzyme.

**TABLE 1:** Kinetic parameters for different substrates of TbCDA determined at 282 nm

Substrate	TbCDA				
	*K_m_* (μM)	*V*_max_ (μmol·min^−1^·mg^−1^)	*K*_cat_ (min^−1^)	*K*_cat_/*K_m_* (μM^−1^·min^−1^)	*V*_max_/*K_m_*
Deoxycytidine	52.3 ± 4.5	34.9 ± 0.8	745.6 ± 17.6	14.3	0.67
Cytidine	199.5 ± 9.0	126.1 ± 3.4	2,690.4 ± 72.0	13.5	0.63
5-Methyl-2′-deoxycytidine	24.5 ± 3.3	5.9 ± 0.2	125.6 ± 4.8	5.1	0.24

**TABLE 2:** Kinetic parameters published for different CDAs

dCtd or Ctd	Result for CDA from^*a*^:											
	*H. sapiens*^*b*^			*A. thaliana*^*c*^			E. coli^*d*^			B. subtilis^*e*^		
	*K_m_*	*V*_max_	*V*_max_/*K_m_*	*K_m_*	*V*_max_	*V*_max_/*K_m_*	*K_m_*	*V*_max_	*V*_max_/*K_m_*	*K_m_*	*V*_max_	*V*_max_/*K_m_*
Deoxycytidine	39	45.5	1.17	75	49	0.65	60	444	7.4	236	230	0.97
Cytidine	39	68.3	1.75	150	59.5	0.39	110	147	1.33	216	184	0.85

a *K_m_* values are expressed in μM. *V*_max_ values are expressed in μmol·min^−1^·mg^−1^.

b See reference 14.

c See reference 13.

d See reference 13.

e See reference 16.

TbCDA activity was also measured for the nucleoside analogue 5-Met-dCtd, giving *K_m_* values on the same order as human CDA (21). 5-Met-dCtd was the substrate for which TbCDA shows the highest affinity, followed by dCtd and Ctd. In contrast, *V*_max_ values were in the order Ctd > dCtd > 5-Met-dCtd. Figure S2 in the supplemental material shows the Michaelis-Menten plots obtained for each substrate assayed.

10.1128/mSphere.00374-19.2 FIG S2 Michaelis-Menten plots for TbCDA kinetic parameter determination for (A) cytidine, (B) deoxycytidine, and (C) 5-methyl-deoxycytidine. The enzymatic assay was performed by incubating 0.125 μM TbCDA with increasing concentrations of cytidine (from 0.025 to 0.4 mM), deoxycytidine (0.025 to 0.4 mM), and 5-methyl-deoxycytidine (0.025 to 0.15 mM) as described in Materials and methods. *V*_max_ and *K_m_* values were calculated by using SigmaPlot v.8 software. Each point represents the average from three independent measurements. Error bars represent standard deviation. Download FIG S2, TIF file, 0.5 MB. Copyright © 2019 Moro-Bulnes et al. 2019 Moro-Bulnes et al. This content is distributed under the terms of the Creative Commons Attribution 4.0 International license.

A comparative analysis of catalytic efficiency (*k*_cat_*/K_m_*) indicated that dCtd is almost as good a substrate as Ctd. The best substrate for dimeric CDAs has been reported to be dCtd, while for human CDA, Ctd is slightly better (13), although other tetrameric CDAs like the enzyme from B. subtilis are more efficient with dCtd (16). There was no evidence of product inhibition.

### TbCDA and not TbdUTPase has a major role in the provision of dUMP for *de novo* thymidylate biosynthesis.

**FIG 2:**
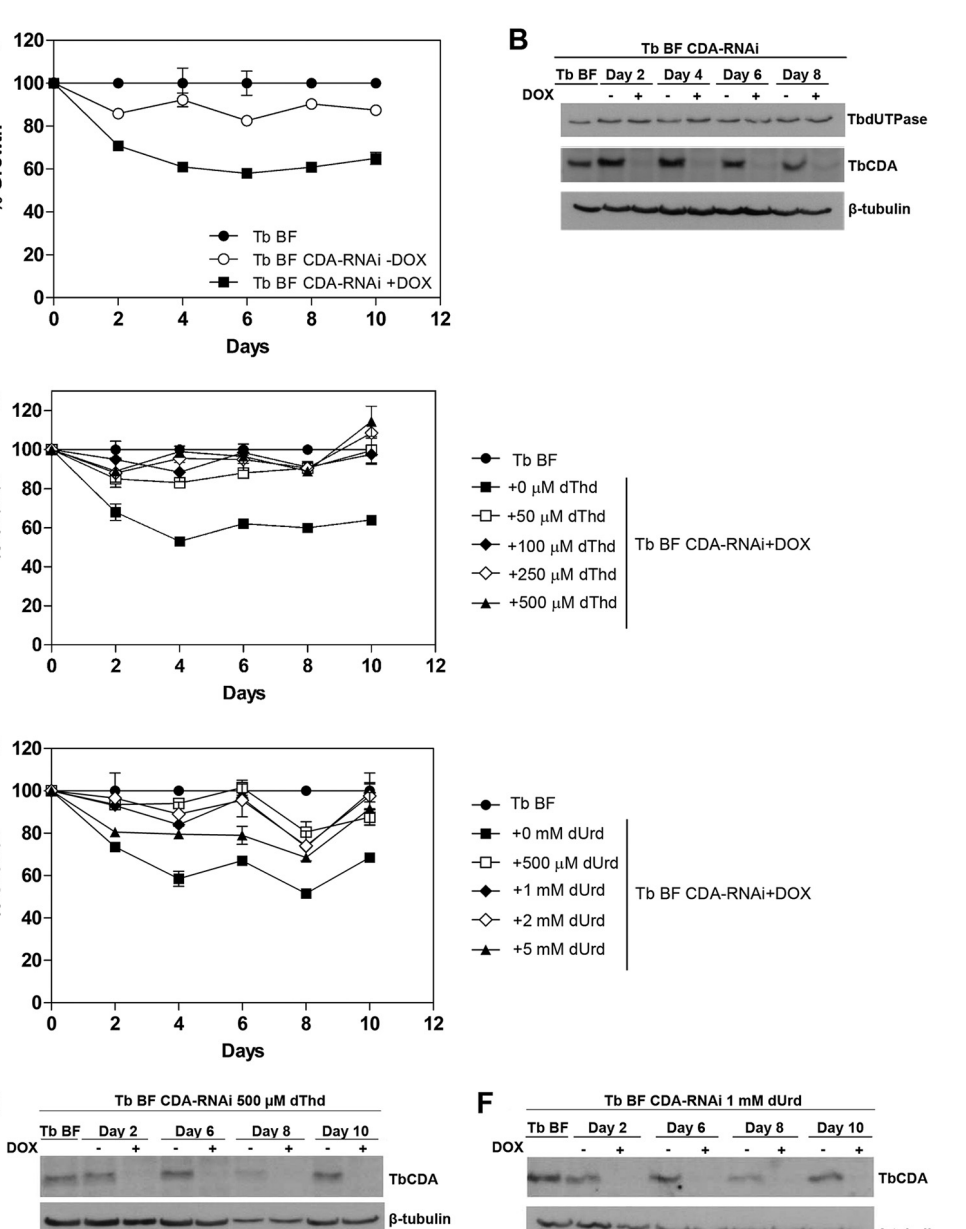
(A) Growth curves of T. brucei BF and BF CDA-RNAi cell lines. DOX, doxycycline. (B) Western blot showing that TbCDA silencing was stable throughout the growth curve and that TbdUTPase levels remain unchanged after 10 days of TbCDA knockdown. (C and D) Growth curve of T. brucei BF and BF CDA-RNAi cell lines supplemented with different dThd (C) and dUrd (D) concentrations. Each point represents the mean from three biological replicates. Error bars represent standard deviations. (E and F) Western blot analysis demonstrating decreased TbCDA expression during supplementation with dThd and dUrd. Anti-TbCDA (1:500), anti-TbdUTPase (1:75,000), and anti-β-tubulin (1:5,000) antibodies were used. A total of 5 × 10^6^ parasites were loaded in each lane.

Previous data (3) have shown that the knockout of TbCDA in bloodstream forms (BFs) gives rise to dThd/dUrd auxotrophy, thus suggesting an essential role in dTMP biosynthesis. The role postulated for TbCDA is the deamination of dCtd to render dUrd, which is subsequently phosphorylated by TbTK to dUMP, the substrate for dTMP biosynthesis. This would be the sole route for provision of dUMP since T. brucei lacks dCMP/dCTP deaminases. In line with this hypothesis, Leija et al. demonstrated that overexpression of human dCMP deaminase (DCTD) in a TbTK-null cell line restores cell growth by directly providing dUMP (3). Here we have performed RNA interference (RNAi)-mediated depletion of TbCDA in T. brucei BF cells (TbBF CDA-RNAi), and in agreement with the observations obtained for CDA-null mutants (3), the depletion of the enzyme resulted in growth defects when grown in HMI-9 pyrimidine-deficient medium (Fig. 2A and B). Supplementation of TbCDA-depleted cells with dThd or dUrd restored growth to levels similar to those obtained in parental cells (Fig. 2C to F), further supporting the role of this enzyme in *de novo* dTMP biosynthesis.

**FIG 3:**
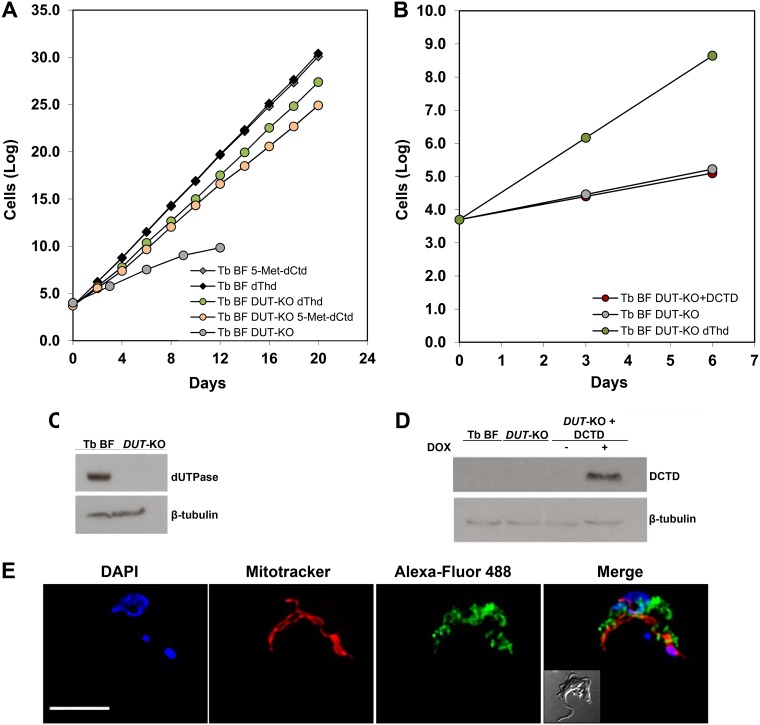
(A) Growth curves of T. brucei BF *DUT*-KO and BF parasites in the presence of dThd or 5-Met-dCtd. (B) Growth curves of T. brucei BF *DUT*-KO in the presence and absence of dThd and of T. brucei BF *DUT*-KO cells expressing human dCMP deaminase (DCTD). Each time point corresponds to the mean for triplicate biological replicates. (C and D) Western blot analyses showing that the T. brucei BF *DUT*-KO cell line lacks dUTPase and that human DCTD is successfully expressed in doxycycline-induced cells. Anti-TbdUTPase (1:75,000), anti-DCTD (1:1,000), and anti-β-tubulin (1:5,000) antibodies were used. Each lane contained 5 × 10^6^ parasites. (E) DCTD subcellular localization in T. brucei BFs expressing human DCTD analyzed by immunofluorescence. Anti-DCTD and Alexa Fluor 488-conjugated goat anti-mouse antibodies were used. Mitochondria were stained with MitoTracker Red CMXRos, and nuclei and kinetoplasts were stained with DAPI. Images were captured using an Olympus IX81 microscope and deconvolved with Huygens Essential software (version 3.3; Scientific Volume Imaging). Images were analyzed by using Fiji software. Bar, 5 μm.

However, T. brucei has a dimeric dUTPase, an essential enzyme that catalyzes the hydrolysis of dUTP/dUDP to dUMP. T. brucei BF dUTPase-null mutants (TbBF *DUT*-KO) have been previously reported to be dThd auxotrophs (Fig. 3A and C) (7). The role of the enzyme has been suggested to be dual: it is involved in the provision of dUMP for dTMP biosynthesis, and on the other hand, it maintains a correct dUTP/dTTP balance and controls uracil incorporation into DNA (7, 8).

The question that hence arises is why cannot TbdUTPase compensate for the deficiency in CDA? If indeed dUTPase has a central role in dUMP production and this is related to its essential character, overexpression of human DCTD in T. brucei BF *DUT*-KO cells grown in thymidine-deficient medium should restore adequate growth by directly providing dUMP. However, notably T. brucei BF *DUT*-KO cells expressing human DCTD died rapidly, in the same fashion as dUTPase-null mutants in the absence of dThd (Fig. 3B and D). The localization of the ectopically expressed enzyme was determined by immunofluorescence to be cytosolic (Fig. 3E). These observations, together with the essential character of TbCDA, support the notion that the main role of TbdUTPase consists in the withdrawal of the excess of dUTP in order to avoid its massive incorporation into DNA rather than contributing to the dUMP pool for dTMP biosynthesis. T. brucei BF *DUT*-KO cells are dThd auxotrophs because the expansion of the dTTP pool would contribute to normalize the dUTP/dTTP ratio, thus avoiding incorporation of dUTP into DNA, an event that is highly cytotoxic (8).

As previously mentioned, the kinetic analysis showed that TbCDA efficiently deaminates the nucleoside analogue 5-Met-dCtd (Table 1). Deamination catalyzed by CDA yields dThd, which theoretically should support growth of *in vitro*-cultured T. brucei BF *DUT*-KO parasites. T. brucei BF *DUT*-KO and parental BF lines were cultured in the presence of either 2 mM 5-Met-dCtd or 600 μM dThd, and parasite growth was monitored for 20 days. As shown in Fig. 3A, the T. brucei BF *DUT*-KO line cultured with 5-Met-dCtd exhibits a comparable growth rate to cells cultured in the presence of dThd. Hence, TbCDA efficiently deaminates pyrimidine nucleoside analogues in T. brucei and has an essential role in the provision of dTMP.

Furthermore, when considering a potential role for dUTPase in providing dUMP for dTMP biosynthesis, it is possible that cross talk may occur between this enzyme and TbCDA. However, TbdUTPase levels remain unchanged in T. brucei BF CDA-RNAi cells (Fig. 2B), reinforcing the supposition that TbdUTPase does not play an important role in dUMP production.

### TbCDA and TbDHFR-TS are located in the mitochondrion of the parasite.

**FIG 4:**
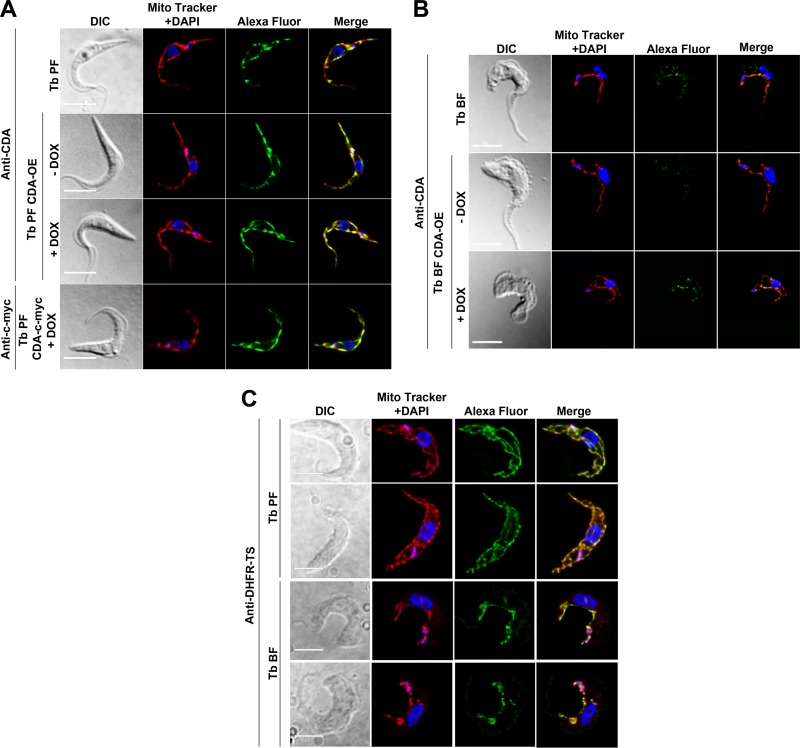
TbCDA and TbDHFR-TS intracellular localization. Immunofluorescence analysis showing the subcellular localization of TbCDA in PF (A) and BF (B) parental cell lines, BFs and PFs overexpressing the enzyme (T. brucei BF CDA-OE and PF CDA-OE, respectively), and PFs overexpressing TbCDA-c-myc (T. brucei PF CDA-cmyc). (C) Immunofluorescence analysis of TbDHFR-TS localization in BFs and PFs. The signal was detected with either anti-TbCDA or anti-TbDHFR-TS and Alexa Fluor 488-conjugated goat anti-rabbit or anti-c-myc and Alexa Fluor 488-conjugated goat anti-mouse antibodies. Mitochondria were stained with MitoTracker Red CMXRos, and nuclei and kinetoplasts were stained with DAPI. Images were captured using an Olympus IX81 microscope and deconvolved with Huygens Essential software (version 3.3; Scientific Volume Imaging). Images were analyzed by using Fiji software. DIC, differential interference contrast. Bar, 5 μm.

The intracellular localization of TbCDA was analyzed by immunofluorescence analysis in both procyclic forms (PFs) (Fig. 4A) and BFs (Fig. 4B) of the parasite using an affinity-purified polyclonal anti-TbCDA antibody. In both cases, for parental cells (upper rows) the enzyme was mostly located in the mitochondrion. In addition, PFs and BFs overexpressing TbCDA were generated together with PFs overexpressing a TbCDA–c-myc fusion protein. Immunofluorescence microscopy using a polyclonal anti-TbCDA antibody and a monoclonal antibody against the c-myc epitope again points toward a major localization of TbCDA in the mitochondrion of the parasite (Fig. 4A and B, lower rows). The colocalization analysis in BFs was performed for the Alexa Fluor 488 and Mito Tracker signals, giving a Pearson’s coefficient of 0.9 ± 0.024. Figure S3 in the supplemental material shows the quantification of TbCDA in both PF (Fig. S3A)- and BF (Fig. S3B)-overexpressing mutants. In addition, Western blot analysis evidenced that the levels of the enzyme in PFs were 2-fold those observed in BFs (Fig. S3C and D).

10.1128/mSphere.00374-19.3 FIG S3 TbCDA fluorescence intensity quantification in both PF (A) and BF (B) parental cell lines and overexpressing mutants. The boxes represent the interquartile range (IQR), and the whiskers are maximum and minimum values. (C) Representative Western blot comparing TbCDA expression levels in PFs and BFs. (D) Western blot quantification of TbCDA expression levels in PF and BF parental cell lines. Error bars represent standard deviation for three biological replicates. Fluorescence and Western blot quantifications were performed using Fiji software. Download FIG S3, TIF file, 1.3 MB. Copyright © 2019 Moro-Bulnes et al. 2019 Moro-Bulnes et al. This content is distributed under the terms of the Creative Commons Attribution 4.0 International license.

To further understand the compartmentalization of dTMP biosynthesis in T. brucei, we performed an immunofluorescence analysis of TbDHFR-TS, the enzyme that subsequently catalyzes the synthesis of dTMP using dUMP as the substrate. A polyclonal anti-TbDHFR-TS antibody obtained in our laboratory was used. Its localization was determined by immunofluorescence in both PFs and BFs of the parasite to be mostly mitochondrial (Fig. 4C). The colocalization analysis performed for the Alexa Fluor 488 stain and Mito Tracker signals gave a Pearson’s coefficient of 0.89 ± 0.012 in T. brucei BFs.

## DISCUSSION

**FIG 5:**
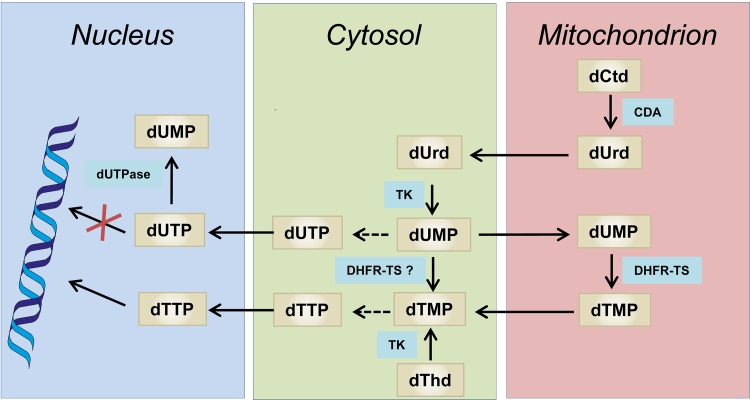
Intracellular compartmentalization of dTTP synthesis in T. brucei. Black arrows indicate the reaction catalyzed by each enzyme. Dashed black arrows refer to a series of reactions catalyzed by several enzymes that may occur in the cytosol. CDA, cytidine deaminase (Tb927.9.3000); DHFR-TS, dihydrofolate reductase-thymidylate synthase (Tb927.7.5480); TK, thymidine kinase (Tb927.10.880); dUTPase, deoxyuridine 5′-triphosphate nucleotidohydrolase (Tb927.7.5160).

Here we have performed different studies aimed at resolving the contribution of TbCDA and TbdUTPase to dUMP and dTMP formation. We have characterized recombinant TbCDA as an enzyme exhibiting deaminase activity on both Ctd and dCtd and have corroborated its essential character by means of RNAi-mediated depletion of enzyme levels. In agreement with previous results in TbCDA knockout cells (3), dThd and dUrd rescue the defective growth of CDA-deficient cells. Based on the observations described in the present study and taking into account available data regarding the intracellular localization of enzymes involved in dTMP biosynthesis, we suggest that dTTP biosynthesis in BFs occurs as depicted in Fig. 5. The figure was assembled using the information available in TrypTag (www.tryptag.org) (22, 23) regarding epitope tagging and intracellular localization of different enzymes involved in dTTP biosynthesis, together with the observations obtained in the present study and published information. TbCDA efficiently deaminates dCtd to dUrd mostly within the mitochondrion since we show using immunofluorescence microscopy that the enzyme is predominantly present in this organelle. On the other hand, we have previously shown that TbdUTPase is nuclear (8), while in the present study we also provide evidence that high levels of TbDHFR-TS are present in the mitochondrial matrix. Altogether the scheme proposed involves the movement of pyrimidine nucleosides and nucleotides in and out of the mitochondrion as indicated. We propose that mitochondrial dCtd is deaminated to dUrd within the mitochondrion. dUrd is transported to the cytosol and nucleus, where it undergoes phosphorylation by TbTK, which is present in both the nuclear and cytosolic compartments (4). The resulting dUMP is methylated to dTMP by TbDHFR-TS and subsequently phosphorylated by thymidylate kinase (TbTMPK) and nucleoside-diphosphate kinase (TbNDPK) to dTTP. TbTMPK (http://www.tryptag.org/?id=Tb927.8.3510) and TbNDPK (24) have been described to occur in the cytosol in procyclics, although a nuclear localization for TbNDPK has also been suggested (24). The scheme assumes that the mitochondrion is mostly permeable to dUrd and dUMP. In addition, dNTPs would be able to cross the mitochondrial and nuclear membranes in order to satisfy the requirements for kinetoplast and nuclear DNA replication. The role of the mitochondrion in dTMP biosynthesis has been previously documented in other organisms. Mitochondria are a major site for folate and dTMP synthesis in plants (25), which also contain a bifunctional DHFR-TS, similar to parasitic protozoa (26), and a *de novo* dTMP biosynthesis pathway has been identified in human mitochondria which contain a novel mitochondrial dihydrofolate reductase (27). In addition, it is interesting to highlight that T. brucei lacks a serine hydroxymethyl transferase (SHMT), the enzyme responsible for completing the dTMP cycle. It has been proposed that in this parasite, mitochondrial 5,10-methylene-tetrahydrofuran (THF) is obtained by the glycine cleavage pathway (28). Mitochondrial formate could be reconverted into formyl and methylene THF cofactors by the trifunctional C1-THF synthase. Indeed T. brucei has a single C1-THF synthase (Tb927.7.1600) that has been predicted to be located in the mitochondrion (http://www.tryptag.org/?id=Tb927.7.1600), thus further justifying the localization of enzymes involved in dUMP biosynthesis in this organelle. With regard to TbdUTPase, previous studies did not allow the pinpointing of the exact contribution of the enzyme to dUMP formation for dTMP biosynthesis. The RNAi-mediated downregulation of the enzyme did not result in perturbed dTTP levels (8), although null mutants were dThd auxotrophs. However, the recent studies performed by Leija et al. (3), together with the data presented here, strongly suggest that the route for dUMP formation via TbTK is essential and that TbdUTPase cannot compensate for the lack of dUrd formation and phosphorylation to dUMP. Altogether, we propose that TbdUTPase, which appears to be located in the nucleus, would be solely responsible for the maintenance of the dUTP/dTTP ratio, thus controlling dUTP incorporation during replication, while TbCDA and TbTK are uniquely involved in dUMP formation necessary for dTMP biosynthesis. The present information highlights singular aspects of dTMP synthesis in T. brucei that may be taken into account in the design of novel therapeutic strategies for the treatment of trypanosomal diseases.

## MATERIALS AND METHODS

### Overexpression and purification of recombinant CDA from T. brucei.

The coding sequence for CDA of T. brucei was amplified by PCR from genomic DNA from the T. brucei *brucei* 427 strain using a pair of gene-specific primers, 5′-GCC ATA TGC AAT GCT CAT CAT TGA AGA CC-3′ (NdeI restriction site underlined) and 5′-GCG GAT CCT CAG AAA GTT CCT GGA CAC C-3′ (BamHI restriction site underlined), designed using the sequence found in the TriTryp database (Tb927.9.3000) and cloned in the pET-28a expression vector (Novagen), which allows an N-terminal His tag fusion protein to be obtained, yielding the pGRV182 construct. Induction of TbCDA was performed in E. coli BL21(DE3) cells harboring pGRV182 with 1 mM IPTG (isopropyl-β-d-thiogalactopyranoside) when an optical density at 600 nm of 0.6 was reached; the culture was incubated for 4 h at 37°C. Cells from 2 liters of culture were harvested by centrifugation at 8,000 rpm for 15 min at 4°C and stored at –80°C until use. For purification, the cell pellet was resuspended in buffer A (20 mM potassium phosphate, pH 7.4, 0.5 M NaCl, 20 mM imidazole) supplemented with a cocktail of protease inhibitors (Complete Mini, EDTA-free protease inhibitor cocktail tablets; Roche). Cells were lysed by sonication and centrifuged at 13,000 rpm for 30 min at 4°C, and the soluble fraction was loaded on a 1-ml HisTrap column connected to a fast protein liquid chromatography (FPLC) system (Pharmacia LKB Biotechnology). After washing, protein was eluted using an imidazole gradient from 20 mM to 1 M at a flow rate of 0.25 ml⋅min^−1^. Fractions containing TbCDA were pooled, and buffer was exchanged with 50 mM Tris-HCl (pH 7.5) using a PD-10 column (GE Healthcare).

### TbCDA enzymatic assays.

Cytidine deaminase activity was assayed spectrophotometrically by measuring the decrease in absorbance at 282 nm, as previously described (14). A concentration of 0.125 μM TbCDA was incubated in 50 mM Tris-HCl (pH 7.5) plus 1 mM dithiothreitol (DTT) with the different substrates assayed (cytidine [Sigma; D-3897], deoxycytidine [Sigma; C-4654], and 5-methyl-2′-deoxycytidine [Santa Cruz Biotechnology; sc-278256]) in a total volume of 1 ml with a rapid kinetics accessory (Hi-Tech Scientific) attached to a spectrophotometer (Cary 50), which was in turn connected to a computer for data acquisition and storage. Protein concentration was determined by the method of Bradford (29).

### Size exclusion chromatography.

The molecular weight (MW) of recombinant TbCDA was determined by gel filtration using a Superdex 200 column (GE Healthcare) connected to an FPLC system (AKTApurifier; GE Healthcare). The column was equilibrated and eluted with 50 mM phosphate buffer (pH 7.4) plus 150 mM NaCl. The following molecular weight markers (Sigma) were used: cytochrome *c* (MW, 12.4 kDa), carbonic anhydrase (MW, 29 kDa), bovine serum albumin (MW, 66 kDa), alcohol dehydrogenase (MW, 150 kDa), β-amylase (MW, 200 kDa), and blue dextran (MW, 2,000 kDa). Samples of 500 μl of either TbCDA or the appropriate protein markers were injected into the column. The size of the subunit was analyzed on 12% polyacrylamide gels by SDS-PAGE. Proteins were stained with Coomassie blue.

### Antibody generation.

Polyclonal antibodies against TbCDA and TbDHFR-TS were generated by immunizing rabbits with denatured and purified recombinant TbCDA or TbDHFR-TS proteins. Four inoculations of ∼300 μg of protein were carried out in a mixture of phosphate-buffered saline (PBS) and Freund´s adjuvant (1:1 ratio). The anti-TbCDA or anti-TbDHFR-TS sera were then collected and affinity purified using homogeneous recombinant protein coupled to Affi-Gel 10 gel (Bio-Rad) resin, following the manufacturer’s instructions.

### Generation of cell lines.

With the purpose of downregulating TbCDA via RNAi, a fragment of 518 bp was amplified by PCR (positions 316 to 549 of the coding sequence and positions 1 to 256 of the 3′ untranslated region [UTR]) using the following primers: 5′-GCG GAT CCA AGC TTG CTG ATG CCA CAG TCC CTC C-3′ (BamHI and HindIII restriction sites underlined) and 5′-GCG TTA ACG GGC CCC ATC CCT GCT GCT CAT TCA C-3′ (ApaI and HpaI restriction sites underlined). The fragment was digested and cloned into the HindIII and ApaI sites of pGR19 (30), and then the same fragment was digested with BamHI and HpaI and cloned in the previous construct in an antisense orientation, yielding pGRA3. Transfection of BFs with this construct yielded the T. brucei BF CDA-RNAi cell line.

In order to overexpress TbCDA, specific primers were used to amplify the coding sequence: 5′-GCC ATA TGC AAT GCT CAT CAT TGA AGA CC-3′ (NdeI restriction site underlined) and 5′-GCG GAT CCT CAG AAA GTT CCT GGA CAC C-3′ (BamHI restriction site underlined). The PCR product was then cloned into the pGRV23b plasmid (31), yielding pGRV192, which was used to transfect both BF (TbBF CDA-OE) and PF (TbPF CDA-OE) cell lines. Additionally, to overexpress TbCDA fused to a c-myc tag, the coding sequence without the stop codon was amplified using the following primers: 5′-GCC ATA TGC AAT GCT CAT CAT TGA AGA CC-3′ (NdeI restriction site underlined) and 5′-GCG TTA ACG AAA GTT CCT GGA CAC CTT GAG TG-3′ (HpaI restriction site underlined) and cloned into the pGRV33 construct (31), yielding pGRV193, which was used to transfect PFs, generating the T. brucei PF CDA-c-myc cell line.

With the purpose of overexpressing human DCTD, total RNA obtained from HeLa cells was used for RT-PCR, and the resulting cDNA was amplified by PCR with a specific pair of primers, 5′-GGA TCC CAT ATG AGT GAA GTT TCC TGC AAG-3′ (NdeI restriction site underlined) and 5′-GTC ATC GGA TCC TCA CTG AAG CTT TTG ACT CGG-3′ (BamHI restriction site underlined), and subsequently cloned into the pGRV23b plasmid (31) to yield pGRV23b+DCTD. Transfection of T. brucei BF *DUT*-KO cells (7) with this construct rendered the T. brucei BF *DUT*-KO+DCTD cell line.

### Trypanosome growth and transfection.

Both the T. brucei single-marker bloodstream form (32) and the procyclic form cell line 449 (33) were used, which were cultured in HMI-9 that contains 80 μM thymidine (or in the case of pyrimidine-deficient experiments, HMI-9 without thymidine) supplemented with 10% (vol/vol) fetal bovine serum at 37°C and 5% CO_2_ and in SDM-79 plus supplemented with 10% (vol/vol) fetal bovine serum and 7.5 μg·ml^−1^ hemin at 28°C, respectively.

Transfections were carried out by electroporation in Cytomix buffer for BFs or Zimmerman buffer for PF cells mixed with 5 to 10 μg of NotI-linearized plasmidic DNA as previously described (32, 34), using a BTX ECM 630 electroporator. The selection drugs used were 5 μg⋅ml^−1^ hygromycin for pGRA3 (BF) and puromycin at 0.1 μg⋅ml^−1^ (BF) or 1 μg⋅ml^−1^ (PF) for pGRV192, pGRV193, and pGRV23b+DCTD. Resistant cells were induced by addition of doxycycline (1 μg⋅ml^−1^).

### Western blot studies.

Cells (5 × 10^6^) for sample preparation were harvested by centrifugation and washed in PBS (137 mM NaCl, 4 mM Na_2_HPO_4_, 1.7 mM KH_2_PO_4_, 2.7 mM KCl). Pellets were resuspended in urea cracking buffer (6 M urea, 10 mM Na_2_HPO_4_, 1% β-mercaptoethanol, pH 7) and boiled after the addition of loading sample buffer (67.5 mM Tris-HCl, pH 6.8, 3% SDS, 10% glycerol, 5% β-mercaptoethanol). SDS-PAGE was performed, and polyvinylidene difluoride (PVDF) membranes were incubated with rabbit polyclonal anti-TbCDA (1:500) antibody generated against recombinant T. brucei CDA, rabbit polyclonal anti-TbdUTPase (1:75,000) antibody generated against recombinant T. brucei dUTPase, or mouse monoclonal anti-DCTD (1:1,000 [Santa Cruz Biotechnology]) or anti-β-tubulin (1:5,000 [Sigma]) antibodies. Bound antibodies were revealed by using goat anti-rabbit IgG (1:5,000) or goat anti-mouse IgG (1:3,000) antibodies (Promega) and the ECL enhanced chemiluminescence detection kit (Amersham Pharmacia Biotech).

### Immunofluorescence studies.

Intracellular localization of DCTD, TbCDA, and TbDHFR-TS was studied by immunofluorescence analysis. Log-phase parasites were harvested, and after 15 min of incubation with MitoTracker Red CMXRos (Invitrogen), parasites were mounted on poly-l-lysine-coated slides, fixed with 4% *p*-formaldehyde at room temperature in wash solution (1× PBS, 0.2% Tween 20) for 20 min, washed, and then blocked and permeabilized with 1% blocking reagent (Roche) containing IGEPAL 0.1% for 75 min. Next, the immunofluorescence assay was performed using either mouse monoclonal anti-DCTD (1:100 [Santa Cruz Biotechnology]), rabbit polyclonal anti-TbCDA (1:100 for PF and 1:50 for BF), rabbit polyclonal anti-TbDHFR-TS (1:1,000 for PF and 1:500 for BF), or mouse monoclonal anti-c-myc (1:100 [Sigma]) primary antibodies in blocking solution. Alexa Fluor 488 goat anti-rabbit (1:40 [Sigma]) or Alexa Fluor 488 goat anti-mouse (1:40 [Sigma]) was used as the secondary antibody. The slides were dehydrated in methanol for 1 min and mounted with Prolong Gold antifade reagent with DAPI (4′,6-diamidino-2-phenylindole [Invitrogen]). Vertical stacks of 30 to 40 slices were captured using an Olympus wide-field microscope and Cell-R IX81 software. Images were deconvolved and pseudocolored with Huygens Essential software (version 3.3; Scientific Volume Imaging). Images were analyzed by using Fiji software (version 1.5e; ImageJ) (35). Colocalization analysis was performed by using the JACoP plug-in (36) calculating the Pearson’s correlation coefficient. Given values are the mean of values from at least 20 cells.
