# Supplementary figures and images for: Contribution of Cytidine Deaminase to Thymidylate Biosynthesis in Trypanosoma brucei: Intracellular Localization and Properties of the Enzyme
Source: mSphere. 2019 Aug 7;4(4):e00374-19. doi: 10.1128/mSphere.00374-19 (PMC6686228; doi:10.1128/mSphere.00374-19)

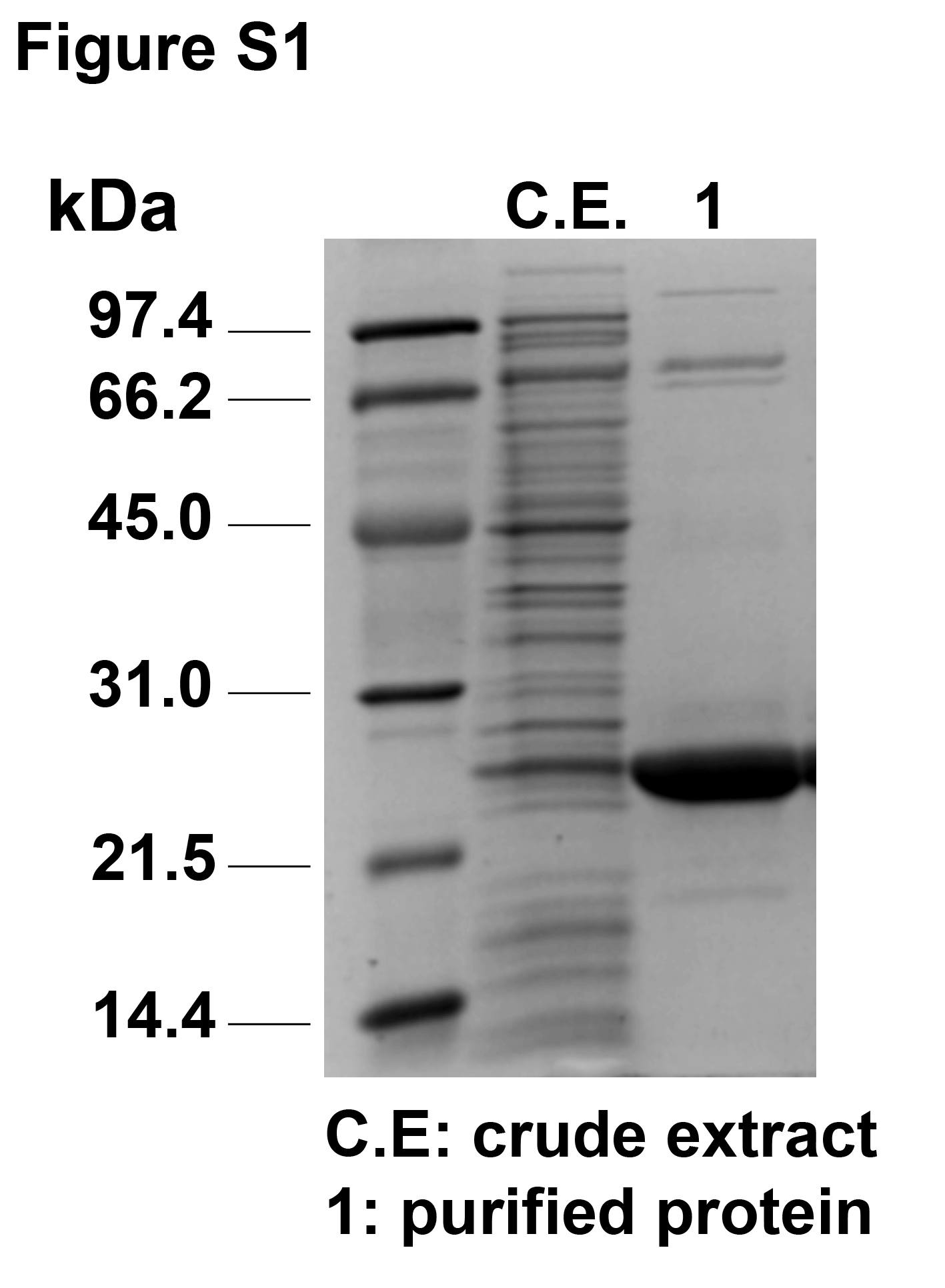

Supplement: FIG S1 [file mSphere.00374-19-sf001.tif]

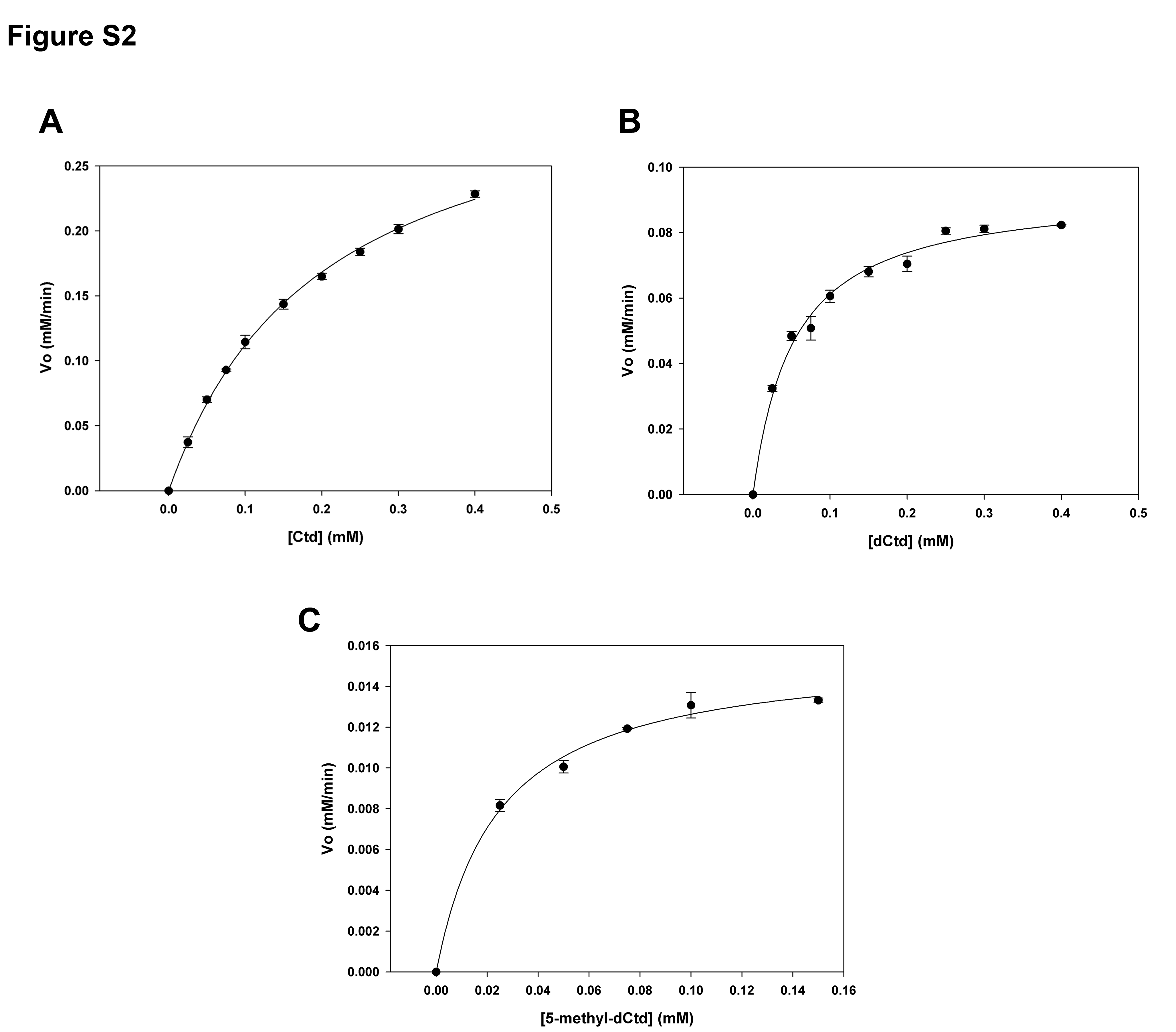

Supplement: FIG S2 [file mSphere.00374-19-sf002.tif]

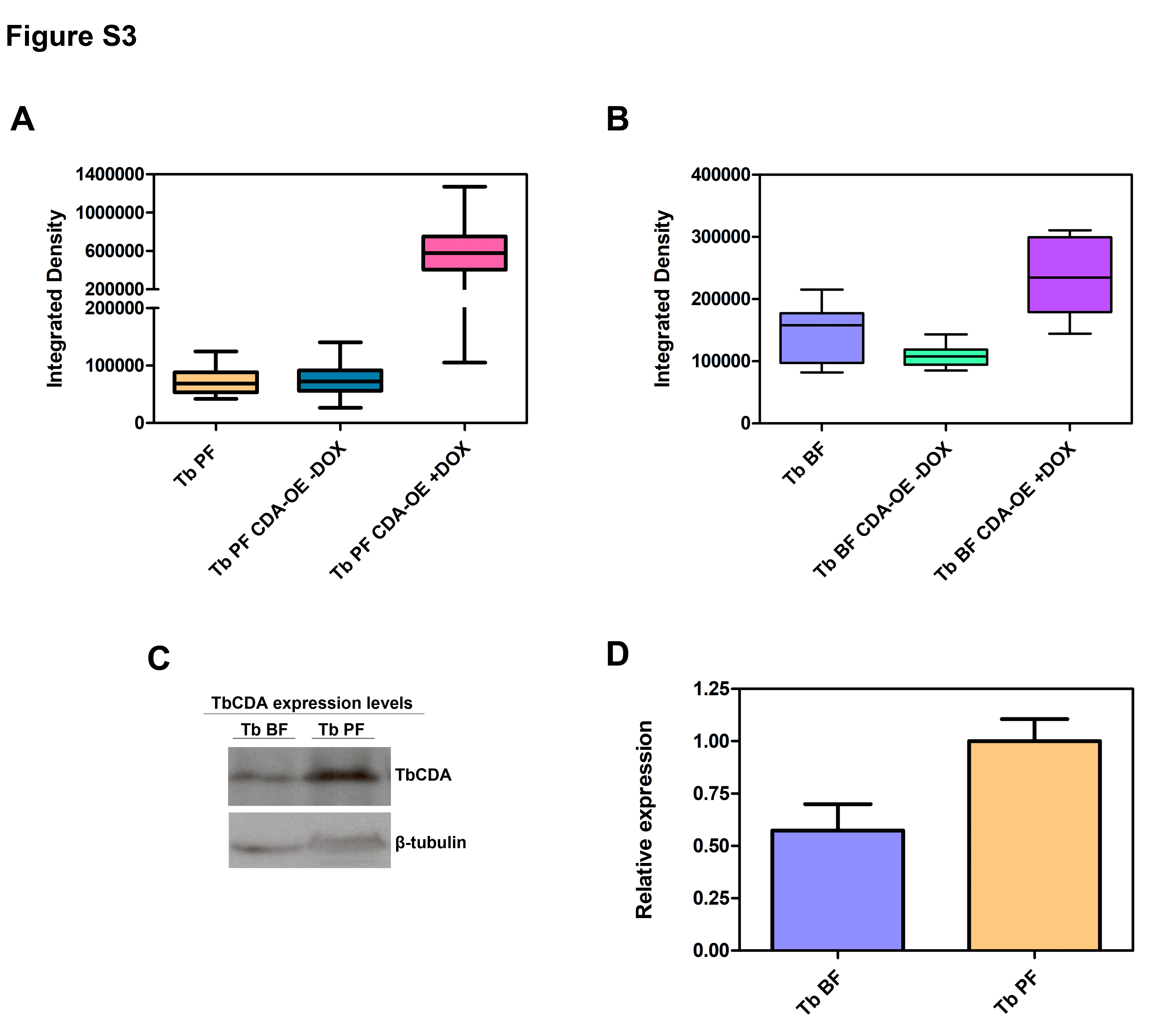

Supplement: FIG S3 [file mSphere.00374-19-sf003.tif]
