# Supplementary material for: Contribution of Cytidine Deaminase to Thymidylate Biosynthesis in Trypanosoma brucei: Intracellular Localization and Properties of the Enzyme
Source: mSphere. 2019 Aug 7;4(4):e00374-19. doi: 10.1128/mSphere.00374-19 (PMC6686228; doi:10.1128/mSphere.00374-19)
Supplement: TABLE S1 [file mSphere.00374-19-st001.pdf]

TABLE S1 purification of recombinant TbCDA

Purification step	Total protein (mg)	Specific activity (µmol/min·mg)	Total activity (µmol/min)	Yield (%)
Crude extract	235.80	0.18	41.66	100
Hitrap+PD10	11.50	22.70	261.05	16
